# BLyS/APRIL dual inhibition by telitacicept for refractory pemphigus vulgaris combined with hepatocellular cancer^☆^

**DOI:** 10.1016/j.abd.2025.501263

**Published:** 2026-01-15

**Authors:** Weiyu Chen, Jian Xu, Yamin Zhang

**Affiliations:** a Department of Dermatology, Tongji Medical College, Union Hospital, Huazhong University of Science and Technology, Wuhan, China; b Hubei Engineering Research Center of Skin Disease Theranostics and Health, Wuhan, China; c Institute of Hematology, Tongji Medical College, Union Hospital, Huazhong University of Science and Technology, Wuhan, China

Dear Editor,

Pemphigus is a rare but potentially life-threatening autoimmune blistering disorder. Corticosteroids and immunosuppressive agents have been the conventional therapy. While some severe variants always fail to respond sufficiently.^1^ Here, we presented a refractory pemphigus case combined with cancer, treated with telitacicept, a novel bioagent simultaneously targeting BLyS (B lymphocyte stimulator) and APRIL (a proliferation-inducing ligand),^2^ achieving complete remission without tumor progression or secondary skin infections.

A woman in her 70 s had been diagnosed with pemphigus vulgaris from another hospital for 2-years, characterized by recurrent oral mucosal erosions, which had been effectively controlled with long-term oral prednisone (10 mg, Qd) treatment. Her skin histopathology and direct immunofluorescence were consistent with the diagnosis of pemphigus vulgaris, while indirect immunofluorescence using rat bladder epithelium didn’t reveal IgG deposition (Fig. 1). She had a medical history of chronic hepatitis B, high blood pressure, diabetes mellitus, and multiple cavity effusions. Six months ago, she was given a diagnosis of hepatocellular carcinoma and received a curative left hepatectomy. The patient was doing well without any evidence of recurrence after surgery. Two months ago, she abruptly presented with severe blisters, erosions, exudations, and crusts, mainly affecting the waist, abdomen, inguinal, and vulva, and was admitted to our ward (Fig. 2). On admission, her pemphigus disease area index (PDAI) score was 21, and a culture of skin exudation revealed the presence of Proteus mirabilis. Given the patient's manifestations of infection, multiple antibiotics were administered. Despite receiving methylprednisolone (1.5 mg/kg/d), intravenous immunoglobulin (400 mg/kg/d, 5-days), meropenem, and plasma transfusion treatments for three weeks after admission, the lesions didn't improve. New blisters and purulent exudation appeared in her groin and back. As the initial therapy failed to control disease progression and combined with liver cancer, telitacicept (160 mg, once weekly) was added, which included systemic corticosteroids (methylprednisolone, 32 mg, Qd), but not anti-CD20 monoclonal antibodies mediated depletion of B-cells, considering that cancer and infections as its absolute contraindications. Following the addition of telitacicept, the patient's erythema, crusts, and exudation began to resolve, and the blister fluid was absorbed after three weeks of treatment. The disease activity was re-evaluated as mild (PDAI = 0) six weeks after being admitted to our ward. Furthermore, after four months of telitacicept treatment, no new blisters were observed, and the dose of methylprednisolone was gradually reduced to 12 mg/day, and telitacicept was reduced to a dose of 160 mg every two weeks. Enzyme-linked immunosorbent assay (MBL, Japan) revealed significantly reduced levels of both Desmoglein (Dsg) 1 and 3 (Fig. 3). No secondary skin infections or liver cancer progression were recorded.

**Fig. 1:**
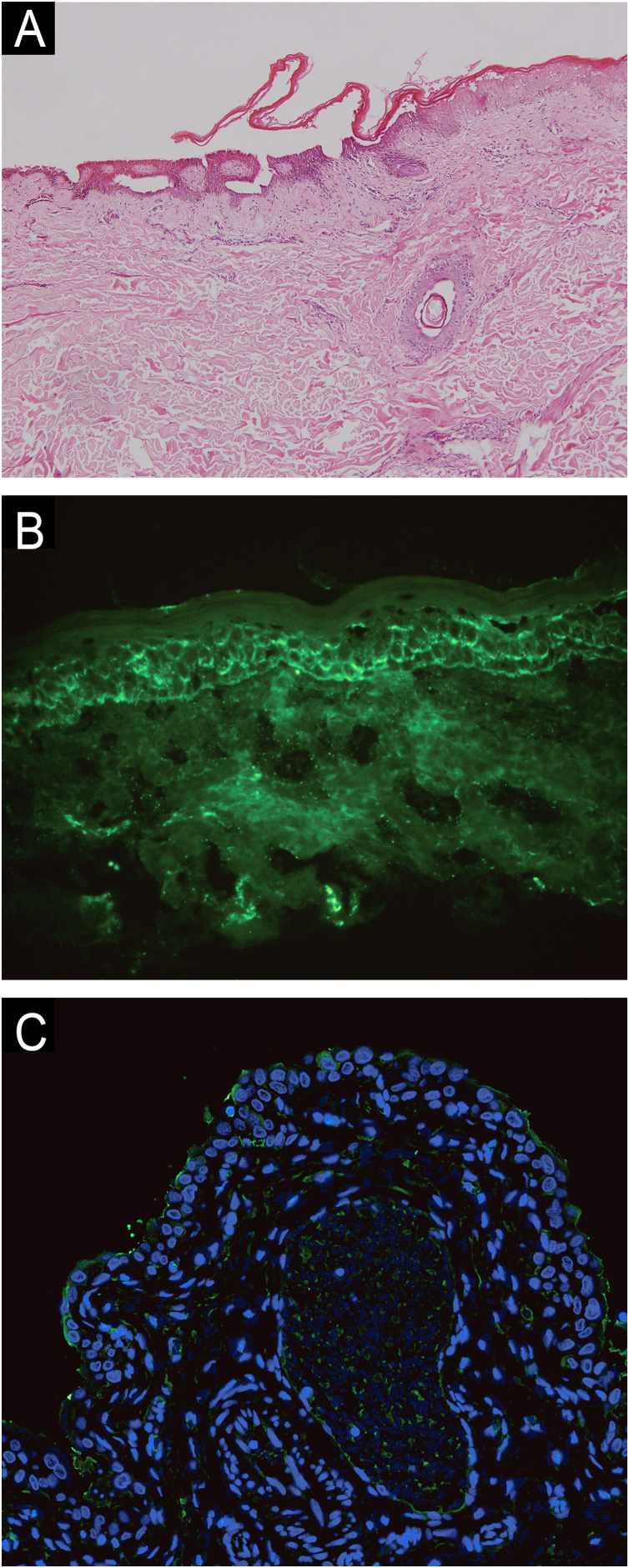
(A) Histopathological examination on the abdomen lesions at first (Hematoxilyn & eosin, 100×). (B) Direct immunofluorescence showing intercellular deposition of IgG in the epidermis (400×). (C) Negative indirect immunofluorescence on a rat bladder epithelium (serum dilution 1:20, 40×).

**Fig. 2:**
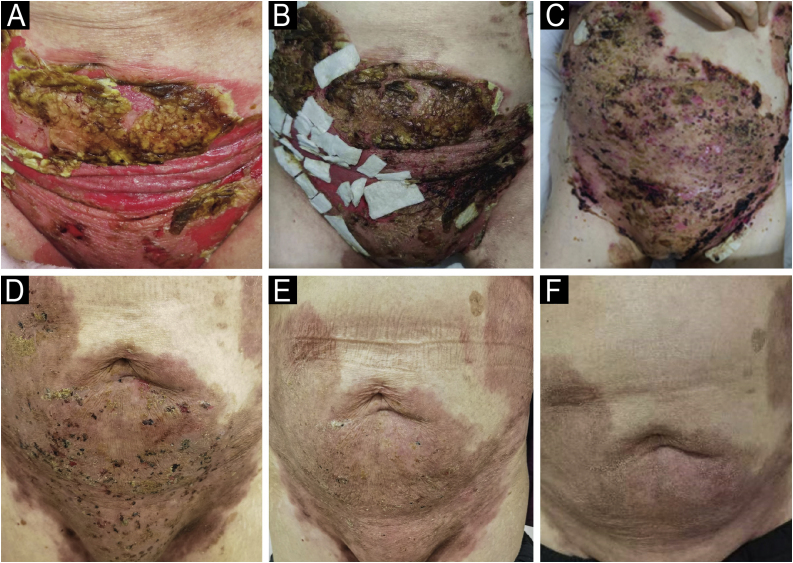
Clinical presentation in refractory patient of pemphigus vulgaris before and after telitacicept therapy. (A) On admission, (B) 2-weeks, (C) 3-weeks, (D) 6-weeks, (E) 10-weeks, (F) 18-weeks.

**Fig. 3:**
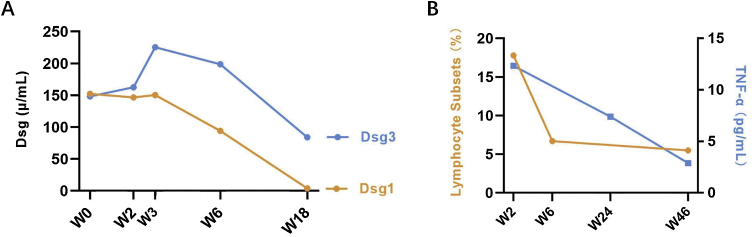
(A) Serum Dsg1 and Dsg3 levels in refractory patient of pemphigus vulgaris treated with telitacicept (Enzyme-Linked Immunosorbent Assay, MBL, Japan). (B) Lymphocyte subsets and TNF-α levels in refractory patient of pemphigus vulgaris treated with telitacicept. The quantification of lymphocyte subsets were done with Automated Hematology Analyzer (mindray, C-760 CS). The TNF-α level was detected by human TNF-α (Tumor Necrosis Factor Alpha) ELISA Kit.

In order to further interpret the reasons why the refractory case of pemphigus vulgaris responded positively to telitacicept without tumor progression or secondary skin infections, we analyzed the potential mechanisms of related drugs. The mechanism of telitacicept targets both BAFF and APRIL, largely eliminating long-lived plasma cells that have developed into potential autoantibody-producing cells and reducing autoantibody levels. However, the anti-CD20 antibodies mediate depletion of peripheral B cells but not long-lived plasma cells and may further exacerbate secondary infection.^3^

Unexpectedly, the lipid levels were found to be elevated, with LDL rising from 1.30 mmoL/L to 1.53 mmoL/L and TG level from 1.25 mmoL/L to 3.71 mmoL/L after telitacicept treatment. The same trend was seen in another two patients who also received telitacicept therapy (data not shown) and other literature published to date of which reported receiving telitacicept therapy (Table 1), although no adverse reactions causing elevated blood lipids were noted in Phase III clinical trials.^4^ Further research is required to better understand the association between these findings.

**Table 1:** Reported cases in the literatures with the use of telitacicept.

Year	Diseases	Sample size	Age (y)	Sex (F/T)	Previous therapies	Concomitant therapies	Time for improvement (w)	Adverse events	Change from baseline in laboratory parameters to predict good prognosis		
									Cells subsets	Immunoglobulin	Others
2023 ^5^	IgG4-RD	9	51.5 median (ranged from 25 to 71)	2/9	A prospective single-arm clinical trial	None	4 (other 8 patients arrive a partial remission rate of 60%)	Injection site reaction, elevated LDL, elevated TG, lymphopenia	CD24- plasmablasts	igg4, igg, iga, igm, ige	C3, C4, Cr, egfr
2022 ^6^	IgAN	44	37.0 (SD = 8.55)	21/44	Systemic corticosteroid therapy, other immunosuppressant therapy	Details unmentioned	4	Injection site reactions, upper respiratory tract infection	None	iga, igg, igm	Proteinuria, egfr
2023 ^7^	GPA	1	64	0/1	Methylprednisolone sodium succinate, immunoproteins	CYC, prednisone	1	Upper respiratory tract infection	None	iga, igg, igm	Crp, esr, crea, bun
2023 ^8^	MN	1	50	0/1	CYC, prednisone, rituximab, MMF, tacrolimus	Prednisone	4	Unmentioned	CD20 cells	None	Proteinuria, egfr, serum albumin
2023 ^9^	pSS	42	49.4 median	40/42	Hydroxychloroquine sulphf	Details unmentioned	4	Local injection reaction, acute pyelonephritis, leukopoenia, infection	None	iga, igg, igm	C3, c4
2024 ^10^	MG	2	64‒70	1/2	Ivig, plasma exchange, prednisone, tacrolimus, pyridostigmine, thymectomy	Prednisone, tacrolimus, pyridostigmine	2	Unmentioned	Memory B cells, plasmablasts, plasma cells	Igg	APRIL, blys, BCMA, achr-Ab
2023 ^11^	NF155 + AN	1	14	1/1	MMF, rituximab, plasma exchange, steroid, immunoadsorption	Details unmentioned	1	Unmentioned	Plasmablasts	None	Anti-nf155

y, year; F, Female; T, Total; w, week; Ref., Reference; IgG4-RD, IgG4-Related Disease; IgAN, IgA Nephropathy; GPA, Granulomatous Polyangiitis; MN, Membranous Nephropathy; pSS, Primary Sjögren's Syndrome; MG, Myasthenia Gravis; NF155 + AN, NF155+Autoimmune nodopathy; CYC, Cyclophosphamide; MMF, Mycophenolate Mofetil; IVIg, Intravenous Immunoglobulin; LDL, Low Density Lipoprotein; TG, Triglyceride; eGFR, Estimated Glomerular Filtration Rate; Cr/CREA, Creatinine; CRP, C-Reactive Protein; ESR, Erythrocyte Sedimentation Rate; BUN, Blood Urea Nitrogen; BCMA, B-Cell Maturation Antigen; AChR-Ab, Acetylcholine Receptor Antibody.

As a new promising biological agent, telitacicept has expanded multiple therapeutic fields besides systemic lupus erythematosus, and varied laboratory parameters were identified to predict its therapeutic response. To summarize clinical characteristics of patients after telitacicept therapy, we conducted a literature review, and the details were summarized in Table 1.^5^, ^6^, ^7^, ^8^, ^9^, ^10^, ^11^ We found that some of the refractory cases whose pathogenesis are related to B-cells and autoantibody levels responded positively to telitacicept. Furthermore, in our case, a downward trend was noticed in her lymphocyte subsets and TNF-α level (Fig. 3), which can be released by B-cells,^12^ and may contribute to the prediction of therapy response. Consistent with our results, in the IgG4-related disease, patients who presented with better therapeutic response to telitacicept had relatively higher levels of serum immunoglobulin and plasmablast at baseline. All the data indicated that B-lymphocyte subsets, inflammatory cytokine, and antibody secreted by B-cells provided a potential predictor for the therapy response, although more cases needed to be intaken.

To the best of our knowledge, this study represents the first exploration of the therapeutic potential of BLyS/APRIL-targeting biologics in treating refractory cases of pemphigus vulgaris accompanied by skin infection and cancer. It also showed a good safety profile for telitacicept, used to treat elderly patients with tumors. Larger sample size studies are necessary to optimize dosage and duration in the future.

## ORCID ID

Weiyu Chen: 0009-0009-6192-4067

Jian Xu: 0009-0005-3762-492X

## Ethics statement

The patients in this manuscript have provided written informed consent to publication of their case details.

## Financial support

This work was supported by grants from the National Natural Science Foundation of China (82003366) and the National Natural Science Foundation of China (82000223).

## Authors’ contributions

Yamin Zhang: Contributed to the conception and design of the study; Contributed to obtaining, analyzing and interpreting data; Engaged in intellectual participation in the propaedeutic and/or therapeutic management of the studied cases; Conducted a critical review of the literature; Provided final approval of the manuscript's final version.

Weiyu Chen: Participated in data collection, as well as the analysis and interpretation of data; Performed the statistical analysis; were involved in writing the article or critically reviewing its important intellectual content; Contributed to obtaining, analyzing and interpreting data; Effectively participated in supervising the research; Engaged in intellectual participation in the propaedeutic and/or therapeutic management of the studied cases.

Jian Xu: Participated in data collection, as well as the analysis and interpretation of data; Performed the statistical analysis; was involved in writing the article or critically reviewing its important intellectual content; Contributed to obtaining, analyzing, and interpreting data; Effectively participated in supervising the research.

## Research data availability

Does not apply.

## Conflicts of interest

None declared.
